# 

**Published:** 1895-01

**Authors:** 


The Association of Erie Railway Surgeons will hold its annual meeting at the Tod House, Youngstown, O., on Tuesday, January 8, 1895, under the presidency of Dr. C. B. Kibler, of Corry, Pa. Dr. C. M. Daniels, of Buffalo, Dr. Henry Flood, of Elmira, and Dr. C. S. Parkhill, of Hornellsville, are among those from the State of New York who will read papers.

---

The Medical Society of the County of Erie will hold its annual meeting in the parlors of the Buffalo Academy of Medicine on

Tuesday, January 8, 1895, at 10 o'clock A. M., under the presidency of Dr. W. H. Gail, of East Aurora. An interesting program is preparing.

---

THE Medical Society of the State of New York will hold its eighty-ninth annual session at the City Hall, Albany, beginning Tuesday, February 5, 1895, under the presidency of Dr. George Henry Fox, of New York.

---

THE Southern Surgical and Gynecological Association scored another success at its Charleston meeting, held on November 13, 14, 15, 1894, under the presidency of Dr. Cornelius Kollock, of Cheraw, S. C. Both the scientific and social features of the meeting were all that could be desired. The scientific work is being catalogued in the journals and merits careful reading. In the course of the president's address he remarked as follows:

It is not surprising that this association should, from its organization and management, prove a success. It has been presided over in the past by men of influence and ability, fitted to shine in any medical circle and to adorn and give dignity to any reunion. Yet not to its presiding officers alone has its success been due. Its founders are earnest men, but there is one among them especially earnest, having its welfare much at heart; a hard and willing worker, an excellent operator, well known by reputation to the medical men of the South and of the North—striving for the advancement of the South, Southern in sympathy, yet catholic in spirit. This man is Dr. W. E. B. Davis, of Birmingham, Ala., the secretary of the association.

The following are officers-elect for the ensuing year: President, Dr. Louis McLane Tiffany, of Baltimore; First Vice-President, Dr. Ernest S. Lewis, of New Orleans; Second Vice-President, Dr. Manning Simons, of Charleston; Secretary, Dr. W. E. B. Davis, of Birmingham; Treasurer, Dr. Richard Douglas, of Nashville. Executive Council—Drs. George J. Engelmann, Wm. D. Haggard, Bedford Brown, Lewis S. McMurtry.

The next session of the association will be held in Washington City, beginning on the second Tuesday of November, 1895.

---

THE editors of *Mathews's Medical Quarterly* desire to obtain the names of all reputable surgeons in the United States, who limit their practice to diseases of the rectum. Address Editors of *Mathews's Medical Quarterly*, P. O. Box 434, Louisville, Ky.

THE *North Carolina Medical Journal* announces that it will appear as a semi-monthly magazine beginning with January, 1895.

It will be published on the 5th and 20th of each month, will be increased in size eight pages a month, and will don a complete new dress, while its subscription price will remain the same.

---

BULLETINS number 6 and 7 of the Harvard Medical Alumni Association are interesting to all graduates of that college and also possess matters of interest to every medical student and physician. The first contains a catalogue of the Alumni and officers together with the constitution. The second comprises a report of the last annual business meeting held June 26, 1894, and a full report of the annual dinner, at which addresses by Dr. W. W. Keen, of Philadelphia, and Dr. William Osler, of Baltimore were made.

---

THE Medical News' Visiting List, one of the best in the market, again presents itself to notice and for the favor of the profession. This book for 1895 follows closely its predecessors in style, make-up and contents, hence need not be described anew at this time. It is, as usual, published in four styles, and the price in any style is $1.25.

Special rates are offered in combination with the *American Journal of Medical Sciences*, the *Medical News* and the Year-Book of Treatment—either one or all. Published by Lea Brothers & Co., 706 Sansom street, Philadelphia.

---

MRS. JANE C. STORMONT, widow of Dr. David W. Stormont, of Topeka, Kan., has contributed $10,000 to assist in the purchase of a site and the erection of a building which, when completed, shall be a hospital for women. It is to be called the Jane C. Stormont Hospital and Training-School for Nurses.

This is the second time Mrs. Stormont has opened her purse to establish medical philanthropic institutions in Topeka. In the issue of the JOURNAL for June, 1892, we noted the fact that she had given $10,000 to establish a medical library. This second gift of Mrs. Stormont is even more praiseworthy than the first.

It is the intention of the hospital board to erect a building of modern architecture and containing every late improvement. A staff of seven physicians will have complete control and management of the hospital and patients. It is probable that Dr. M. B. Ward will be appointed surgeon-in-chief.

---

SEND twenty cents in stamps to McArthur Hypophosphite Company, Boston, for McArthur Vest-Pocket Diary, 1895. "Handiest and most useful little book for the physician that we have seen. Contains doses of drugs, including new remedies, a list of disinfectants and how to use them, antidotes for poisons, methods of treating emergencies, an obstetric ready reckoner and other valuable information."—*Brooklyn Medical Journal.*

---

THE attention of our readers is invited to the initial advertisement of Schulze-Berge & Koechl, which appears in our columns in this issue. This firm represents in this country that well-known and extensive chemical industry, the Farbwerke vorm. Meister Lucius & Brüning, Hoechst-on-the-Main, Germany, and they have already successfully placed before the profession such standard medicinal products as antipyrin, dermatol, lanolin and tuberculin (Koch). They will also handle in the United States the new diphtheria antitoxin (Behring). Information and descriptive literature concerning the newer remedies which they import, will be cheerfully furnished when requested.

---

THE New York Pharmaceutical Company, of Bedford Springs, Mass., advertises for the first time in the JOURNAL in this issue. Their card appears on advertising page xxx., to which attention is invited.

---

NOTICE—A CALENDAR FOR 1895.—Upon receipt of request, P. Blakiston, Son & Co., Medical Booksellers, 1012 Walnut street, Philadelphia, will send free by mail, postage prepaid, a neat desk calendar for 1895.

---

AN EXCELLENT temperature chart, prepared by Dr. D. T. Laine and published by W. B. Saunders, 925 Walnut street, Philadelphia, has the usual rulings in squares for tracings and ample space for memoranda giving the daily history of each case. On the back is printed a synopsis of the method of Brand in the treatment of typhoid fever.

These charts are sold in pads of twenty-five at fifty cents a pad.

---

The Columbia Calendar, published by the Pope Manufacturing Co., has paid us its annual visit. The issue for 1895 is especially attractive in its make-up. With new dress and new thoughts for every day in the year, it is a useful piece of desk furniture and is a daily reminder of the popularity of the Columbia bicycle, which is manufactured by this company at Hartford, Conn. The branch office in Buffalo is at 609 Main street.

---

The Memorandum Calendar, published by the Davidson Rubber Co., Boston, Mass., is especially intended for physicians and druggists, but is useful on everybody's desk. Its separate leaflets for every day in the year, to be torn off each morning, contain also notices of the Davidson rubber goods, whose worth is everywhere acknowledged. Stoddart Brothers, 84 Seneca street, are among the agents announced for these popular goods.

---

The *Index Medicus* will, it is stated, cease to be published with the February number, owing to the lack of support and the delinquency of a large number of its subscribers, unless an effort is made to continue it.

It will be possible to continue the *Index Medicus* if 500 new subscribers are obtained. The subscription price is $10 per annum, which should be sent to Mr. George S. Davis, publisher of the *Index Medicus*, Box 470, Detroit, Michigan.

---

The *Journal of Medicine and Science* is the name of a new journal, published at Portland, Me., which made its first appearance in December. It is the official organ of the Maine Academy of Medicine, that has lately been organized. It is edited by Dr. E. E. Holt, who is assisted by Dr. J. A. Spaulding, and the names of the officers and Fellows of the Academy appear as collaborators.

---

The New York Central and Hudson River Railroad, the only line in the world owning 450 miles of four tracks, may be said to be the great sanitary railway. This claim is based on the grounds of speed, safety, ventilation and cleanliness.

These points of excellence appeal to physicians. Its advertisement will be found on page xxii. of the Journal.

